# Two-dimensional arrays of vertically packed spin-valves with picoTesla sensitivity at room temperature

**DOI:** 10.1038/s41598-020-79856-0

**Published:** 2021-01-08

**Authors:** Marilia Silva, Fernando Franco, Diana C. Leitao, Susana Cardoso, Paulo P. Freitas

**Affiliations:** 1 Instituto de Engenharia de Sistemas e Computadores - Microsistemas e Nanotecnologias (INESC-MN), 1000-029 Lisbon, Portugal; 2 Instituto Superior Tecnico (IST), Universidade de Lisboa, 1040-001 Lisbon, Portugal; 3 INL - International Iberian Nanotechnology Laboratory, 4715-330 Braga, Portugal; 4 Present Address: Analog Devices, Limerick, V94 RT99 Ireland

**Keywords:** Spintronics, Electronic and spintronic devices, Sensors and biosensors, Magnetic properties and materials

## Abstract

A new device architecture using giant magnetoresistive sensors demonstrates the capability to detect very low magnetic fields on the pT range. A combination of vertically packed spin-valve sensors with two-dimensional in-plane arrays, connected in series and in parallel, delivers a final detection level of 360 pT/$\sqrt{Hz}$ at 10 Hz at room temperature. The device design is supported by an analytical model developed for a vertically packed spin-valve system, which takes into account all magnetic couplings present. Optimization concerning the spacer thickness and sensor physical dimensions depending on the number of pilled up spin-valves is necessary. To push the limits of detection, arrays of a large number of sensing elements (up to 440,000) are patterned with a geometry that improves sensitivity and in a configuration that reduces the resistance, leading to a lower noise level. The final device performance with pT detectivity is demonstrated in an un-shielded environment suitable for detection of bio-signals.

## Introduction

**Figure 1:**
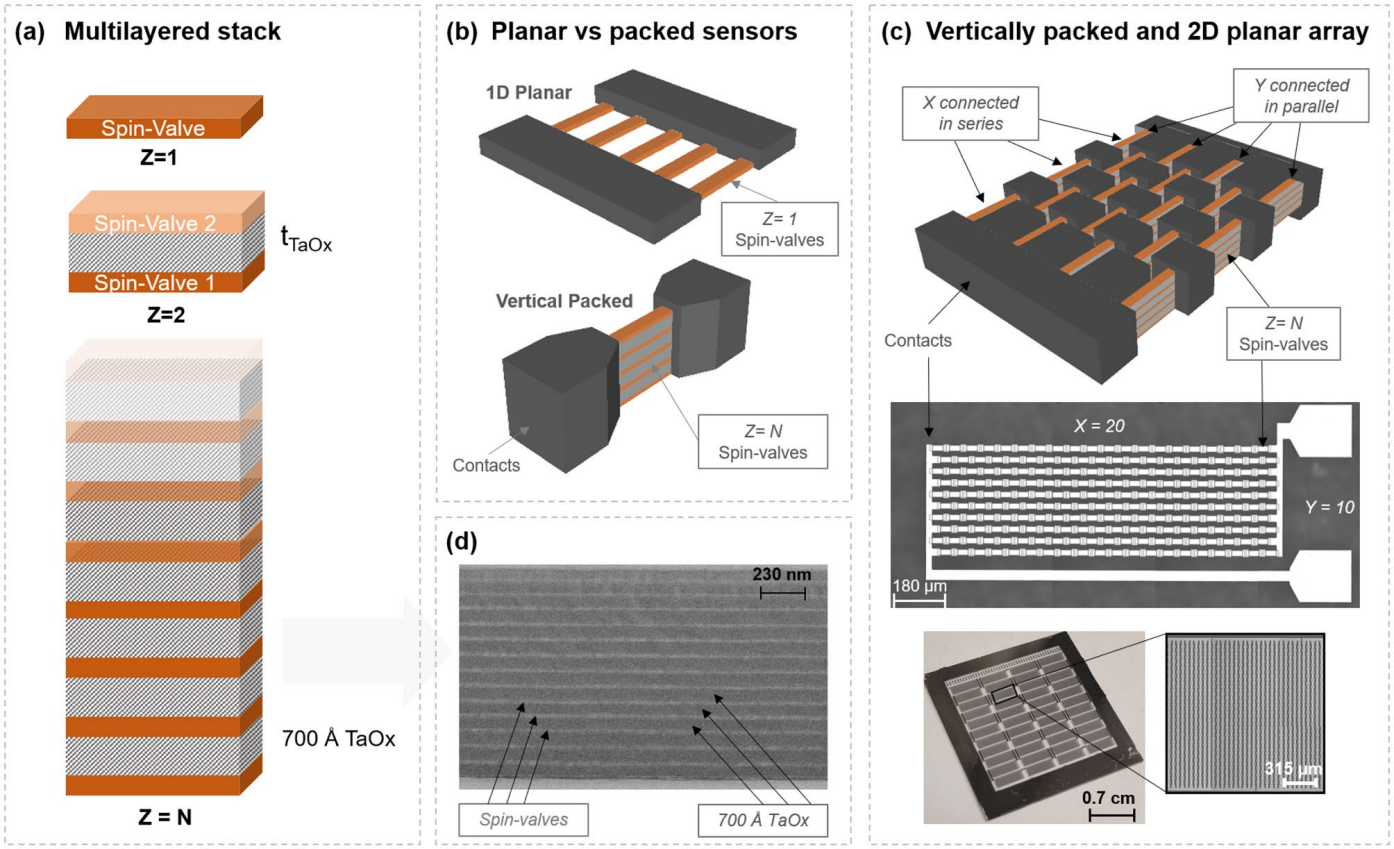
Designs of the spin-valve arrangements considered in this study. (**a**) Scheme of vertically packed multilayered thin-films with $Z=1$ for single spin-valve, $Z=2$ for two vertically packed spin-valves with different TaOx spacer thicknesses (from 500 to 1100 Å), and $Z=5,10,N$ for multiple vertically packed spin-valves with fixed TaOx thickness of 700 Å. (**b**) Scheme of one-dimensional planar array versus a vertically packed array; both have the elements connected in parallel. (**c**) Scheme of vertically packed (*Z*) two-dimensional (*XY*) array of spin-valves ; optical images of patterned device containing 1000 sensors ($X=20$, $Y=10$, $Z=5$) and the final device with best performance showing 440,000 elements. (**d**) Scanning electron microscopy (SEM) cross sectional image of a $Z=N$ multilayer. The bright layers corresponds to the spin-valves while dark thick layers are the TaOx (700 Å).

Emerging biomedical applications using portable sensor devices offer patients individualized and improved health monitoring^1,2^. High precision instruments for magnetic imaging^3–5^ are contactless and can offer superior outputs than electrical measurements, since magnetic signals are not attenuated by human tissue^6,7^. Current technologies for ultra-low magnetic field detection include Superconducting QUantum Interference Devices (SQUID)^8^ and Optical Pumping Magnetometry (OPM)^9,10^ reaching femto-tesla detection levels and more recently Spin Exchange Relaxation Free (SERF) atomic magnetometers on pico-tesla range^11,12^. Despite the very low field detection capability, SQUIDs require cryogenic environments^13^ and OPMs rely on complex optical parts^14^, hampering a seamless compact and wearable design. Therefore, the next generation of medical devices demands small and robust solutions with low power consumption and high sensitivity^15,16^, compatible with electronic integration^17,18^ and flexible substrates^19^ for enhanced portability and compactness. A reliable solution for compact devices working at a room temperature can be found in magnetoresistive (MR) sensors^20–22^. Several applications of giant-MR based spin-valve sensors show nT detection levels and high spatial resolution^7,23–25^. To push the detectivity further into pT range, thus becoming competitive with magnetic tunnel junctions^26^ or hybrid magnetic-MEMS devices^27^, different solutions were introduced. Large area arrays^28,29^ and their combination with magnetic flux guides^26^ have successfully delivered improved performances, at the expense of lower spatial resolution. Given the maturity of stack engineering for spin-valve multilayered thin films^24,30^, new design strategies are necessary to meet the demands in detection limits. We introduced the concept of a vertically packed arrangement of spin-valve sensors wherein the sensing elements are connected in parallel as seen in Fig. 1a^31^. The overall device resistance was reduced and consequently the noise level. An improved detectivity was demonstrated while maintaining a compact design for better spatial resolution. However, a strong decrease in sensor sensitivity is also observed due to magnetostatic coupling fields acting on the sensing layers^32,33^.

In this work, we present a new solution for an extremely sensitive magnetic device working at room temperature. The sensor architecture combines vertically packed spin-valves separated by an oxide spacer, with an in-plane two-dimensional array of elements (Fig. 1b,c). A new numerical model for packed spin-valves is introduced, accounting for all intra and interlayer magnetic couplings^34^ to support the design of the multilayered sensor. To achieve high field sensitivity in the vertically packed sensors, non-magnetic spacers can be used or sensor physical dimensions can be changed. The impact of each parameter on the magnetic behavior of the sensing device is addressed. The target is to minimize the effects of the magnetostatic coupling fields that directly affect the linear range of the sensors. The optimized final configuration combines sensors connected in series (*X*) and in parallel (*Y*), with vertical packing (*Z*). For an array of 440,000 elements (Fig. 1c), a record detectivity of 360 pT/$\sqrt{{\mathrm{Hz}}}$ at 10 Hz is achieved at room temperature. In an unshielded environment, this device is capable of detecting magnetic fields above 500 pT at low frequencies (35 Hz). With this vertical and planar packing strategy, we push the state-of-the-art in giant magnetoresistive sensors showing improved field detectivity comparable to that of series of magnetic tunnel junction sensors^28^ and becoming more competitive with fluxgate technology^35^ displaying pT detectivity^36–38^ with a few $\hbox {cm}^2$ area. Still, the thin film magnetoresitive technology allows to further enhance the spatial resolution by increasing the number of sensors in vertical direction (Z) while reducing the area in the plane; therefore reaching the requirements for bio signal detection at room temperature such as those needed for magnetocardiography^39,40^ and magnetomyography^5^.

## Results and discussion

Figure 1 shows the fabricated device designs, using spin-valves. A spin-valve is a thin film multilayer structure composed of two ferromagnetic (FM) layers separated by a Cu spacer^16^. In a top-pinned configuration, the top FM layer has its magnetization direction fixed through exchange bias by an adjacent antiferromagnetic layer, while the magnetization of the bottom FM layer is free to rotate when a small magnetic field is applied, hence sensing layer. The current flows in the plane of the device (CIP) yielding a high resistance value when the magnetization of the two FM layers are antiparallel, and a low resistance when they align parallel to each other. Upon microfabrication, these multilayered systems can be engineered so that the sensing layer magnetization changes its direction through coherent spin rotation. A linear transition between the low and high resistance states is thus achieved being suitable for sensing applications. The micropatterned spin-valve is now sensitive to the strength and direction of the magnetic field (vector sensor)^24,41–43^. The full thin-film structure is obtained by alternating the growth of spin-valves and TaOx, a scheme repeated several times for increased number of vertically packed structures (*Z*; Fig.1d). Three groups of multilayered thin-films were prepared: (i) single spin-valve ($Z=1$), (ii) two vertically packed spin-valves ($Z=2$) with different TaOx thickness (from 500 to 1100 Å), and (iii) multiple vertically packed spin-valves ($Z=5,10$) with fixed TaOx of 700 Å. The set with $Z=5, 10$ was then patterned into 2D arrays (Fig. 1c) with different element size.

**Figure 2:**
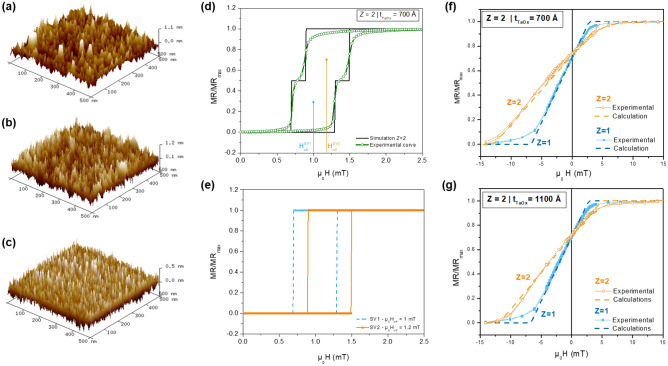
AFM images measured on the top surface of (**a**) a single spin-valve $Z=1$, (**b**) a spin-valve /TaOx 700 Å/spin-valve $Z=2$, and a (**c**) 700 ÅTaOx film. Transfer curves for (**d**, **e**) unpatterned samples and (**f**, **g**) micropatterned sensor. (**d**) Comparison for $Z=2$ of an experimental normalized R(H) transfer curve for a spacer of TaOx 700 Åand the corresponding simulated output. (**e**) simulated curves for each individual spin-valve in the system considering the bottom one ($\hbox {SV}_1$) with lower offset field ($\hbox {H}_{\mathrm{off}}$) than the top one ($SV_2$) with higher offset field. Input parameters for analytical calculations include $H_k$ and $M_{sat}$ values in Table 1. Normalized transfer curves comparing the double packed spin-valves ($Z=2$) with TaOx thickness of (**f**) 700 Åand (**g**) 1100 Å, and comparison with the single spin-valve structures. Experimental data for patterned structures with $w=2\,\upmu m$ and $l=40\,\upmu m$ is compared with the analytic calculations using Eq. (3) and input parameters of Table 1.

### Unpatterned thin-film

Figure 2a–c compares the surface topography of a single spin-valve, a spin-valve/TaOx 700 Å/spin-valve structure and a 700 Å TaOx film. Average roughness values of ${\mathrm{R}}_{\mathrm{a}} = 2.1$ Å, 2.4 Å and 1.2 Å were obtained, respectively. The first value is consistent with literature for these multilayered thin-films^44^. The TaOx roughness of few Å is within optimum range to grow spin-valve structures. The $\hbox {R}_{\mathrm{a}}$ increases slightly from $Z=1$ to $Z=2$ suggesting increased roughness in the top spin-valve structure. A higher offset field (Néel coupling) is expected^44^, consistent with M(H) results obtained for $Z=1$ and $Z=2$ (Fig. S1 from Supplementary Material). Figure 2d shows the normalized transfer curve of the unpatterned $Z=2$ system with TaOx 700 Å. The minimum resistance is $\hbox {R}_{\mathrm{min}} = 13 \, \Omega$, smaller than $\hbox {R}_{\mathrm{min}}= 26 \, \Omega$ for a single spin-valve ($Z=1$), in line with a system connected in parallel. Figure 2e shows the theoretical output obtained for each spin-valve using the macrospin model and considering different $\hbox {H}_{\mathrm{N}}$^45^. Equation (1) shows the total energy for the sensing layer of an unpatterned spin-valve film ($Z=1$):

1 $$E_{total}^{film}=-\mu _0 (\overrightarrow{H}_{app} + \overrightarrow{H}_{N} ) \cdot \overrightarrow{M}_{SL} +K_u \sin^2 \theta . $$

Contributions include the external applied magnetic field ($H_{app}$), the Néel orange-peel coupling field ($H_{N}$) acting on the sensing layers, and the intrinsic uniaxial magnetic anisotropy field ($K_u$) defined during deposition by an applied magnetic field. $M_{SL}$ is the magnetization of the sensing layer^45^. For unpatterned spin-valve films the effect of the stray fields created by the pinned-layer [top $\hbox {Co}_{80}\hbox {Fe}_{20}\,26$ Å], the sensing layer [$\hbox {Ni}_{80}\hbox {Fe}_{20}$ 25/$\hbox {Co}_{80}\hbox {Fe}_{20}$ 28 Å], the self-demagnetizing field due to lateral confinement, and the field created by the flowing current can be neglected. An energy minimum occurs for $\theta = \pi$ for negative external magnetic fields until $\hbox {H}_{\mathrm{ext}} < - \hbox {H}_{\mathrm{N}} +\hbox {H}_{\mathrm{k}}$, and for $\theta = 0$ if $\hbox {H}_{\mathrm{ext}} > - \hbox {H}_{\mathrm{N}} - \hbox {H}_{\mathrm{k}}$, yielding a squared shape (Fig. 2e) where $H_{off}$ = $H_{N}$ and the coercivity $H_{c}$ = $H_{k}$. Figure 2e shows the calculations for $Z=2$ considering decoupled spin-valves . $\mu _0\hbox {H}_{\mathrm{N}} =1$ mT is considered for the bottom spin-valve ($\hbox {SV}_1$) and $\mu _0 \hbox {H}_{\mathrm{N}} =1.2$ mT for the top spin-valve ($\hbox {SV}_2$). For a $Z=2$ unpatterned system the final $MR/MR_{max} (H)$ follows the equivalent resistance formula given by:

2 $$\begin{aligned} \frac{MR}{MR_{max}}=\frac{{M}_{SL}\cos (\theta _1) + {M}_{SL}\cos (\theta _2)}{2}, \end{aligned}$$

where $\theta$ represents the angle between the pinned and sensing layers magnetization. These calculations correlate very well with the experimental MR(H) and justify the observed kink (Fig. 2e). The offset field values agree with the increased roughness observed by AFM.

### Micropatterned sensor: impact of spacer thickness and physical dimensions

Figure 2f,g compares MR(H) of micropatterned $Z=1$ and $Z=2$ spin-valve systems, with TaOx thicknesses of 700 Å and 1100 Å. A MR = 4.5% and a $\hbox {R}_{{min}}= 698$ $\Omega$ is obtained for $Z=1$ and MR = 4$\%$ and $\hbox {R}_{{min}}= 397$ $\Omega$ for $Z=2$ (Fig. S2, Supplementary Information). $Z=1$ shows a non-hysteric behaviour with a linear range of $\mu _0 \Delta H$ = 9.5 mT centered at 1.8 mT. A linear fit within ±0.2 mT around the offset field gives a sensor sensitivity $S=\frac{\Delta MR}{\Delta H}=$ 0.4%/mT. For $Z=2$ an increase of the linear range and the offset field is observed, when compared with $Z=1$. $\Delta H$ increases by more than 80%, to 17.8 mT and 17.1 mT for TaOx = 700 Å and TaOx = 1100 Å, respectively. This corresponds to a decrease of field sensitivity of 55% and 45%. The offset field also increases to 4.1 mT for TaOx = 700 Å and 3.5 mT for TaOx = 1100 Å.

**Figure 3:**
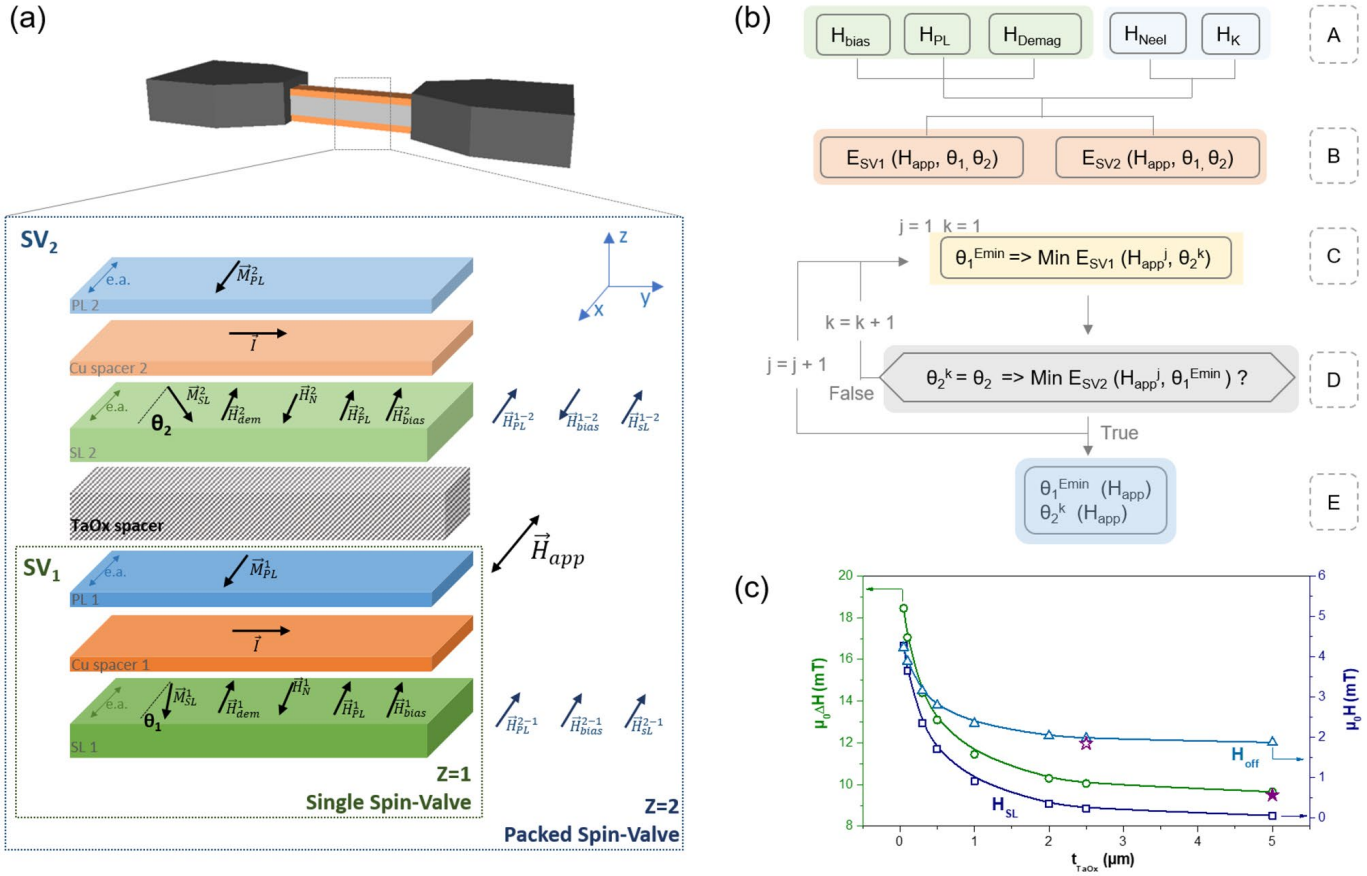
(**a**) Schematic of two spin-valves separated by a TaOx thick spacer for the implementation of the analytical model. ${\mathrm{SV}}_1$ stands for the bottom spin-valve (deposited first) and $\hbox {SV}_2$ is the top spin-valve grown on top of the TaOx spacer. All magnetic field contributions acting on the sensing layers are represented ($H_{app}$, $H_{off}^{1,2}$, $H_{PL}^{1,2}$, $H_{bias}^{1,2}$, $H_{dem}^{1,2}$). Crossed inter-layer couplings are also added, namely the dipolar fields from $\hbox {SV}_2$ pinned layer and sensing layer ($H_{PL}^{2-1}$, $H_{SL}^{2-1}$) acting on $\hbox {SV}_1$ (and vice-versa), and field created from the flowing current $H_{bias}^{2-1}$ (and vice-versa). $M_{SL}^{1,2}$ and $M_{PL}^{1,2}$ are the magnetizations of the sensing (SL) and pinned (PL) layers, respectively. (**b**) Employed algorithm to solve the $Z=2$ spin-valve system. At stage A, the self-demagnetizing and stray fields acting in each SL are calculated, while offset field and induced uniaxial anisotropy are obtained from experimental R(H) curves of unpatterned samples. At stage B two matrices $E_{SV1}$ and $E_{SV2}$ are generated with the total energy of the SL of each $\hbox {SV}_{1,2}$ as a function of external field applied $\hbox {H}_{{app}}$ and all combinations of $\theta_1$ and $\theta_2$. At stage C is initialized a loop to find the $\theta$ that minimize the energy for both spin-valves at a given applied field. Firstly, $\theta_1$ that minimizes the $E_{SV1}$ is found for a $\hbox {H}_{{app}}$ and $\theta _2^k$.Then, at stage D is verified if the conditions at C minimizes simultanously the $\hbox {E}_{{SV2}}$, otherwise the angle $\theta _2$ is swept until both energies are minimized. Consequently, the previous loop restart for a different $H_{app}$ in order to generate the output at stage E. (**c**) Calculated linear range (green; Eq. (3)), sensing layer stray field (blue; Eq. (8)) and offset field (light blue; Eq. (3)) as a function of TaOx spacer thickness for $Z=2$. The star points indicates experimental values for a single SV of $\Delta H$ (closed $\bigstar$) and $H_{off}$ (open $\bigstar$).

A more complex system arises now upon micropatterning, when compared to the one described in Eq. (1). Figure 3a shows a schematic design for $Z=2$ spin-valves that includes all contributions accounted for in this new macrospin model. Magnetostatic interactions between the two spin-valves are now considered, in addition to self-demagnetizing effects, bias current fields and pinned layer stray fields. For the used $H_{app}$ range, the pinned layer magnetization is fixed along the magnetic easy axis (Fig. 3a)^45^.

For each sensing layer (${\mathrm{SL}}_1$ and ${\mathrm{SL}}_2$, Fig. 3a), three inter-layer coupling terms come into play. Acting on ${\mathrm{SV}}_1$ from ${\mathrm{SV}}_2$, are (i) the dipolar field from pinned layer ($H_{PL}^{2-1}$), (ii) the field created by the current flowing on ${\mathrm{SV}}_2$ ($H_{bias}^{j-i}$) and the (iii) dipolar field from sensing layer ($H_{SL}^{2-1}$). Similar reasoning is done for the ${\mathrm{SV}}_2$ system. Due to such couplings, ${\mathrm{SL}}_{1,2}$ magnetization reversal in both spin-valves is not independent. Consequently, the total energy of ${\mathrm{SV}}_1$ (${\mathrm{SV}}_2$) sensing layer is given by the following macrospin model:

3 $$E_{total}^{SV_1} =-\mu _{0} ( {\overrightarrow{H}}_{bias}^{1} + \overrightarrow{H}_{app} + {\overrightarrow{H}}_{N}^{1} + {\overrightarrow{H}}_{PL}^{1}+ \frac{{\overrightarrow{H}}_{dem}^{1}}{2} + {\overrightarrow{H}}_{bias}^{2-1} + {\overrightarrow{H}}_{SL}^{2-1}+ {\overrightarrow{H}}_{PL}^{2-1})\cdot {\overrightarrow{M}}_{SL}^{1} + K_{u}\sin^{2}(\theta ).$$

$H_{dem}^{1}$ is the self-demagnetizing field while $H_{PL}^{1}$ and $H_{bias}^{1}$ are the stray fields originated by ${\mathrm{SV}}_1$ pinned layer and bias current, respectively. The numerical solution of $\theta _1$ and $\theta _2$ in Eq. (3), that minimize the entire packed system energy (i.e. considering simultaneously both sensing layers) follows the algorithm depicted in Fig. 3b. The computational diagram is initialized by computing the total energy of each spin-valve. Then, the unique $\theta _1$ and $\theta _2$ that satisfy the condition of minimum energy for both spin-valves at a certain $H_{app}$ are found. In the end, only a single energy minimum is considered for a linear output response^45^.

The input values for the model are summarized in Table 1. The $H_{N}^{1,2}$, $H_{k}^{1,2}$ and saturation magnetization ($M_{sat}$) for CoFe and NiFe were extracted from unpatterned M(H) curves. A bias current of 1 mA per SV is used, however only 30% is considered to flow through the Cu layer and thus contribute to $\mu _0H_{bias}^1$ = 0.1 mT.

Figure 2f,g compares the calculated output with the experimental curves. The calculated (normalized) MR(H) accurately depicts the experimental data obtained for micropatterned sensors. The significant increase in linear range observed for packed systems is consistent with an inter-layer coupling field that reinforces $H_{dem}$, being the main responsible for the decrease in sensitivity. This arises from the stray fields of $SL_{1,2}$, whose orientation and intensity change according to $H_{app}$. Furthermore, the shift in MR(H) also indicates an additional field parallel to the sensing direction. Crossed contributions such as $H_{PL}^{1-2, \,2-1}$ and $H_{bias}^{1-2, \,2-1}$ reinforce the Neel coupling.

However, the packed system offset field and linear range can reach those of a single spin-valve using a first approach by increasing the spacer thickness while maintaining the high aspect ratio as shown in Fig. 3c. The calculated linear range ($\Delta H$), dipolar field from SL (Eq. (8)) and offset field are here presented as a function of TaOx thickness. A strong $H_{SL}^{1-2, \,2-1}$ is obtained in the low thickness range, vanishing for 5 $\upmu m$. From this point on, each spin-valve in the $Z=2$ system becomes magnetically independent, approaching the limit of a single spin-valve (star point in Fig. 3c). The calculated offset field is dependent on the constant values of $\mu _0H_{PL}^{1,2}$ (2.9 mT), $\mu _0H_{N}$, $\mu _0H_{bias}^{1,2}$ and changing values of $\mu _0H_{PL}^{1-2}$ or $\mu _0H_{PL}^{2-1}$. When the latter becomes negligible ($t _{TaOx}>2.5\, \upmu$m), the $Z=2$ offset field is comparable to a single spin-valve.

**Table 1:** Input parameters used for analytic calculations.

Parameter	*w*	*l*	$\mu _0H_{N}^{1}$	$\mu _0H_{N}^{2}$	$\mu _0H_{k}$	$M_{sat}^{NiFe}$	$M_{sat}^{CoFe}$
Value	2 $\upmu$m	40 $\upmu$m	1 mT	1.2 mT	0.3 mT	930 kA/m	1250 kA/m

**Figure 4:**
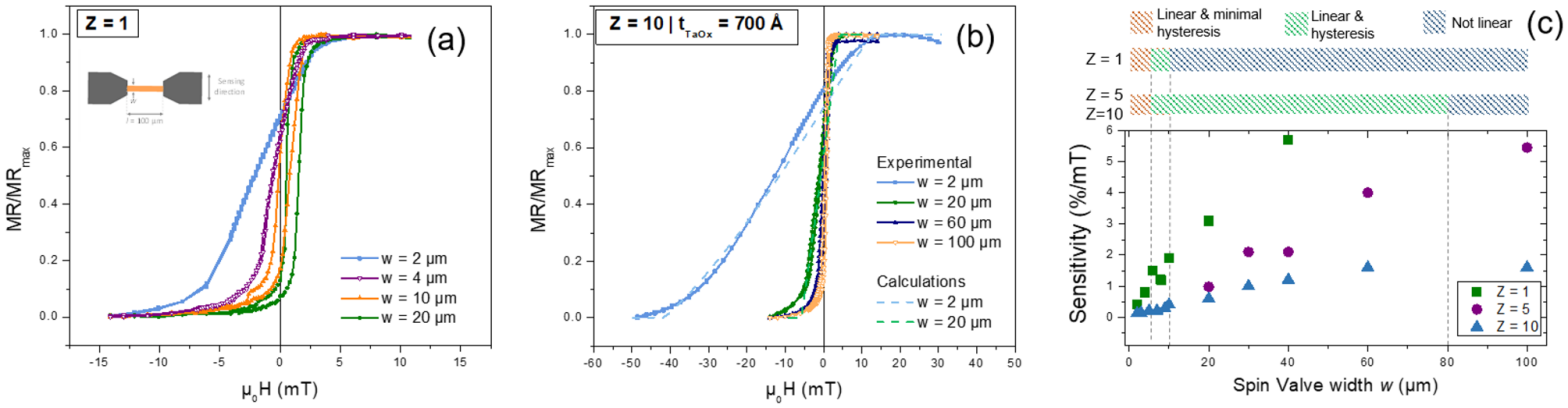
Normalized transfer curve of spin-valve sensor for (**a**) Z = 1 and (**b**) Z = 10 by varying the width from 2 to 100 $\upmu$m. For Z = 10 is also shown calculated curves from the developed macrospin model for w = 2 $\upmu$m and 20 $\upmu$m. (**c**) Sensitivity as a function of the sensor width obtained at the most sensitive point of the curve. Three regions are defined to describe the sensor behavior accounting for its hysteresis and linear output.

As alternative to magnetically decouple the vertically packed spin-valves, long deposition times of a thick spacer layer can be substituted by engineered physical dimensions of the sensor (*w* and *l*). Using the developed macro spin model for double packed spin-valves (Z = 2) and considering a thin spacer layer (e.g. 700 Å), the contributions from the stray fields decrease with increasing *w*, therefore the $H_{SL}^{j-i}$ along the sensor width decreases very sharply until $w=10\, \upmu$m and mostly vanishes above $w=80$ $\upmu$m (see Fig. S3 (a) in Supplementary Information). This effect consequently improves the sensitivity and reduces the offset approaching those of a single spin-valve with fixed $w= 2 \,\upmu$m (see Fig. S3 (b) in Supplementary Information). Furthermore, this strategy can be effectively employed for $Z > 2$. The real impact on the MR(H) curve, of altering the physical width ($w=2$ to 100 $\upmu$m) in packed systems ($Z=5$ and $Z=10$), was experimentally evaluated and compared with calculated curves from the macro spin model for Z = 1 and Z = 10 (Fig. 4a,b). Results from macro spin model for Z = 5 and 10 are shown in Fig. S3 (c)–(h) of the Supplementary Material. Figure 4c compares the sensitivity and hysteresis in all studied structures. Three regions are visible: (i) linear and minimal hysteresis ($\mu _0 \hbox {H}_{\mathrm{c}} < 0.05$ mT), (ii) linear with small hysteresis ($\mu _0 \hbox {H}_{\mathrm{c}} > 0.1$ mT), and (iii) not linear ($\mu _0 \hbox {H}_{\mathrm{c}} > 0.4$ mT) and saturated at zero field.

For $Z=1$, linear outputs are achieved up to $w = 5\,\upmu$m, becoming linear and hysteretic until $w = 10\,\upmu$m and then completely squared. For $Z=5$ and $Z=10$, small hysteresis appears above $w =5\, \upmu$m,loosing the linearity for $w > 80 \,\upmu$m.

In region (i), the sensitivity for $Z > 1$ is lower than $Z=1$ since dipolar coupling and demagnetizing field contribute to enlarge the linear operation range. In region (ii), packed spin-valves are still linear although with a small hysteresis for a broad range of *w* (5 $\upmu$m$< w < 80\, \upmu$m). This translates into a sensitivity similar to that observed for a linear $Z=1$ spin-valve (0.4%/mT). For region (iii), $w >80$ $\upmu$m MR(H) becomes square, as the intrinsic anisotropy dominates, hindering its use as a magnetic field sensor. An additional magnetic field, applied perpendicular to the sensing direction, is effective in reducing this hysteresis but adds complexity to the fabrication of a compact device design (Fig. S4 of Supplementary Material)^26^.

### 2D arrays: reducing noise, increasing detectivity

**Figure 5:**
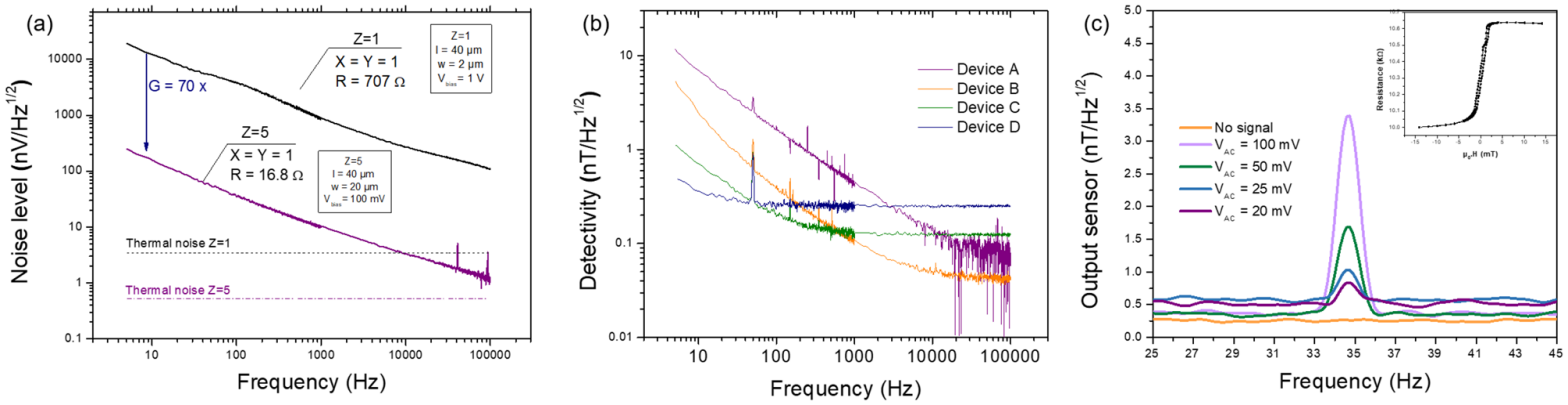
(**a**) Noise curves for a single $Z=1$ and $Z=5$ packed spin-valves, measured at the most sensitive point of the curve ($H_{app}=0$). The two dashed lines indicate the calculated thermal noise baseline. The bias voltage was calculated in order to have about 1 mA flowing per each spin-valve. (**b**) Detectivity curves obtained through the ratio between noise and sensitivity for four devices (Device A, B, C, D in Table 2) with different number of sensors. Device A was biased with 1 V and the others with 3 V. (**c**) Output of Device D under a bias voltage of 3 V, in an unshielded environment and subjected to variable voltage amplitude applied to the external coil at 35 Hz. The inset shows R(H) of Device D with MR = 6.3%, $R_{min}= 10$ $k\Omega$, $\mu _0H_{off}=0.2$ mT, $\mu _0H_{c}=0.25$ mT and a zero field sensitivity of 1.7%/mT (or 17.2 V/V/T).

In spin-valve sensors the main sources of noise are thermal and 1/f noise^29^, being the total noise voltage power spectral density ($S_V$) given by:

4 $$\begin{aligned} S_V^2=4k_BTR+\frac{\gamma V^2}{n_c\,f}, \end{aligned}$$

where $k_B$ is the Boltzmann constant, *T* the temperature, *R* the sensor resistance ($R= R_{\square }\frac{l}{w}$), $\gamma$ the Hooge 1/f noise parameter, *V* the bias voltage, $n_c$ the number of charge carriers ($n_c = V \times C= wltC$), and *f* the frequency. When the system evolves to a packed configuration and its physical dimensions are altered, the noise is given by:

5 $$\begin{aligned} S_V^2=4k_BTR_{\square }\frac{l}{wZ}+\frac{\gamma V^2}{Z\,wltC}\frac{1}{f}. \end{aligned}$$

where *Z* is the number of packed sensors. By vertically stacking sensors with a large area ($w \times l$) and connected them in parallel, a lower resistance is obtained, promoting a general noise reduction over the entire frequency spectrum. Figure 5a compares the noise level for two devices $Z=1$, $w = 2\,\upmu$m and $Z=5$, *w* = 20 $\upmu$m, chosen for their optimal linear output according to the discussion above. Measurements are performed under a bias voltage of 1 V and 100 mV, for *Z* = 1 and 5 respectively, thus ensuring 1 mA per spin-valve.

Consequently, two devices *i* and *j* with different properties (*i*) $Z_i$, $V_i$,$w_i$ and (*j*) $Z_j$, $V_j$,$w_j$ reveal a gain $G_{ji}$ in noise level (ratio between the two $S_V$) at low frequency given by:

6 $$\begin{aligned} G_{ji}=\sqrt{\left( \frac{V_i}{V_j}\right) ^2 \frac{w_j}{w_i}\frac{Z_j}{Z_i}}. \end{aligned}$$

An improvement (decrease) of 70 times in the noise level is obtained for $Z=5$ at low frequencies. Although, the spatial resolution increases by a factor of 10, the decrease in the noise level is more significant.

**Table 2:** Details of the characterized vertically-packed 2D arrays.

Device	A	B	C	D
Lateral size *w* ($\upmu$m)	20			
Vertically packed *Z*	5			
Connected in series *X*	20			
Connected in parallel *Y*	10			
Device A repetitions ($N_A$)	1	10	110	440
Number of sensors	1000	10,000	110,000	440,000
Detectivity @ 10 Hz (${\mathrm{nT/}}\sqrt{\mathrm{Hz}}$)	6.9	2.4	0.71	0.36
Calculated gain @ 10 Hz	1	3.2	10.0	21.0
Measured gain @ 10 Hz	1	2.9	9.7	19.4

*Z* is the number of vertical spin-valves, *X* connected in series and *Y* in parallel. Sensor A represents an array of $X=20$, $Y=10$ and $Z=5$ with a total number of 1000 sensors. Sensor B, C and D are the array A replicated 10,110 and 440 times, respectively. The detectivity gains, calculated and measured, are obtained relative to Device A.

Our approach goes beyond packing sensors in the vertical direction (*Z*)^31–33^, pushing further on the detection level by using in-plane two-dimensional arrays with *X* sensors connected in series and *Y* sensors connected in parallel. The noise level of such *XYZ* array is now given by:

7 $$\begin{aligned} S_V^2=4k_BT\frac{X}{Y\,Z}R+\frac{\gamma X V^2}{YZ\,wltC}\frac{1}{f}. \end{aligned}$$

An overall improvement of $\sqrt{\frac{X}{YZ}}$ is therefore expected for the entire noise spectrum considering the same physical dimensions.

A compromise between the physical dimension and sensitivity exists in packed system, and extends to planar arrays. Comparing a device with $Z = 1$, $X = 40$, $Y = 25$ ($w = 2$ $\upmu$m) and another with $Z = 5$, $X = 20$, $Y = 10$ ($w = 20$ $\upmu$m), a theoretical gain of 6.4×  at low frequency is predicted. Experimentally, a gain of 5.6×  with an improvement in spatial resolution of 1.5×  is achieved. In fact, only by increasing *Z* from 5 to 10, while maintaining physical dimensions and *XY* number of sensors, a larger improvement in spatial resolution of 2× is obtained, although the expected gain in noise is only $\sqrt{2}$ (Fig. S6 in Supplementary Information).

Figure 5b compares the detectivity level for different devices (A to D), consisting of $Z=5$ vertically-packed 2D arrays of sensors with *w* = 20 $\upmu$m (Table 2). The detectivity is the ratio of the sensor’s noise level and its sensitivity (signal-to-noise ratio equal to 1). The choice of geometrical features considers a compromise between spatial resolution and detectivity. $Z=10$ could be used for lower noise, however a large *w* had to be used to ensure linearity and higher sensitivity (see Supplementary Information).

At 10 Hz, Device A shows a minimum detectable field of 6.9 nT/$\sqrt{Hz}$. Since, the detectivity improves by $1/\sqrt{ XYZ}$ (Eq. (7)), pT resolution is possible by increasing the total number of sensors by at least 100 times (gain of 10×). Device A was therefore replicated several times (Devices B, C and D) to achieve the targeted performance (Fig. 1c; Table 2). Due to limitations of the measuring system, Device A was biased with 1 V and the others with 3 V. Nevertheless, at low frequencies (10 Hz) the range for biomedical aplications, the detectivity does not depend on the voltage. The same is not valid for high frequencies where the detectivity varies with $1/\sqrt{V}$. Comparing the performance at 10 Hz, the best detectivity is obtained for Device D (440,000 sensors) with 360 ${\mathrm{pT}}/\sqrt{\mathrm{Hz}}$ and a gain of 19.4×. Table 2 summarizes the results obtained for all devices showing a good correlation between measured gain and calculated gain according to $\sqrt{N}$. The drawback of this strategy is the increase in occupied area, which can be partially compensated by larger *Z*.

### Measurements in an un-shielded environment

Device D is then used to measured a very low magnetic field without magnetic shielding, as in a real experiment environment. Figure 5c shows the magnetic field detected by the device. This is obtained from converting of the voltage output into field units using the device sensitivity. The inset of Fig. 5c shows the R(H) transfer curve biased with 3 V and exhibiting a sensitivity of 17.2 V/V/T. The baseline and limit of detection of 360 pT/$\sqrt{Hz}$ are obtained with a measurement without any AC signal similar for shielded environment. Then a voltage sweep from 100 to 20 mV was applied, resulting in a magnetic field of 3400 pT/$\sqrt{Hz}$ and 800 pT/$\sqrt{Hz}$ for the limits of applied voltage. Due to thermal drifts, an increase in the signal baseline during the measurements is visible. The last measurement shows a SNR = 1.7 (4.6 dB) setting the threshold for signal detection.

## Conclusions

By merging vertically-packed and in-plane arrays of sensors into a highly sensitive device we are able to detect picoTesla fields in an unshielded environment. Optimized packed spin-valve systems are demonstrated, overcoming the loss of sensitivity due to magnetostatic couplings. Two different routes to mitigate dipolar couplings and improve sensitivity are outlined by the implemented numerical model. This new tool encloses all crossed magnetic coupling terms and allowed us to explore their influence in the self-demagnetizing field of the sensing layers for micropatterned double-packed spin-valve architectures. The first approach requires changing the thickness of TaOx spacer while maintaining the high aspect ratio of the sensing elements, setting the threshold for complete magnetic decoupling at 2.5 $\upmu$m spacer thickness which poses challenges for experimental implementation. The second route relies on changing the sensor element physical dimensions (low aspect ratio) while preserving a thin and low roughness spacer. Experimentally, two-dimensional arrays of spin-valve elements with *Z* up to 10, enclosing a thin spacer of 700 Å with low roughness and optimum geometry were successfully fabricated by using standard deposition and micropatterning techniques. An array with five vertically-packed spin-valves and 20 sensing elements connected in series and 10 in parallel replicated 440 times, shows the capacity of measuring pT signals above a detection threshold of 360 pT/$\sqrt{Hz}$ at 10 Hz, fitting the specification for biomedical signal detection.

## Methods

### Growth of vertically packed multilayers

The multilayer thin-films and the insulator spacer were grown with ion beam deposition tools (N3000/N3600). The spin-valve stack used is composed of [Ta 20/$\hbox {Ni}_{80}\hbox {Fe}_{20}$ 25/${\mathrm{Co}}_{80}\hbox {Fe}_{20}$ 28/Cu 24/${\mathrm{Co}}_{80}\hbox {Fe}_{20}$ 26/${\mathrm{Ir}}_{24}\hbox {Mn}_{76}$ 70/Ta 50 (Å)]. The spacer between consecutive spin-valves is tantalum oxide (TaOx). Ta is well known to act as a good buffer for magnetosresistive stacks^46,47^. TaOx was grown by depositing Ta assisted by a non accelerated $\hbox {O}_2$ plasma ($\hbox {V}^{+}=\hbox {V}^{-}= 0$ V; RF power: 160 W, $\hbox {O}_2$ flow: 10 sccm).

#### Sensor microfabrication

The spin-valve thin-films were patterned by photolithography and ion milling etching at 70 degrees to obtain a steep lateral profile along the full thickness. For Z=1 and 2 and varying TaOx thickness, a length of $l = 40\,\upmu$m and width $w = 2\,\upmu$m was used to define the sensor. For Z = 5 and 10 and $\hbox {t}_{{TaOx}}= 700$ Å, the structures were patterned with $l = 40\,\upmu$m and 100 $\upmu$m, changing *w* from 20 to 100 $\upmu$m. Planar arrays ($Z=1$) and vertically packed arrays ($Z=5$ and 10) were fabricated with the number of sensors connected in series (*X*) changing from 10 to 40, and connected in parallel (*Y*) from 2 to 25 sensors (Fig. 1c). Electrical contacts were defined by liftoff of $\hbox {Al}_{98.5}\hbox {Si}_{1.0}\hbox {Cu}_{0.5}$ 3000/${\mathrm{Ti}}_{12.5}\hbox {W}_{50}\hbox {N}_{37.5}$ 150 [Å] and passivated with $\hbox {Al}_2\hbox {O}_3$ 1000 Å. Annealing was then performed at $250^{\circ }\hbox {C}$ for 30 minutes, and the sample cooled down under an external magnetic field of 1 T.

#### Characterizations

Unpatterned films were characterized by Vibrating Sample Magnetometry (VSM) and electrically using four point probe measurement. Scanning Electron Microscopy (SEM) was performed in a Raith 150 Ebeam/SEM system. Atomic Force Microscopy (AFM) was performed to access the impact on topography from different TaOx spacer thicknesses. Areas of 500 nm by 500 nm were scanned, with a frequency of 1 Hz and 256 lines.

The patterned structures were measured using a DC two-point probe method with 1 mA bias current within a field range of ±14 mT. The sensor noise spectra were acquired within a frequency range of dc-100 kHz at room temperature on a magnetically shielded box. The device under test is biased through a battery to mitigate the 50 Hz power network component while the output is amplified 100× by a low noise amplifier SIM910 that is connected to a spectrum analyzer.

The evaluation of the limits of detection in an unshielded environment used a low resistance Helmholtz coil (N=8 and r=2.5 cm) connected to a 10 $k\Omega$ resistor. The circuit is biased with a AC signal with a voltage changing from 100 mV to 20 mV at a frequency of 35 Hz to create an AC magnetic field (0.34 pT/nA). Each measurement was done with a gain of 100 through a low noise amplifier and averaged 50 times within a resolution bandwidth (RBW) of 1 Hz between 5 and 200 Hz.

#### Calculation of the demagnetizing field

For the model considered in Eq. (3), only uniformly magnetized media are assumed, and thus a macroscopic description using magnetostatic equations is chosen to calculate $H_{dem}^{1,2}$, $H_{PL}^{1,2}$ and crossed terms $H_{PL}^{1-2}$, $H_{PL}^{2-1}$ and $H_{SL}^{1-2}$, $H_{SL}^{2-1}$^48^:

8 $$\begin{aligned} \overrightarrow{H}_{r}= \frac{1}{4 \pi } \int _S \frac{\overrightarrow{n \cdot \overrightarrow{M} (\overrightarrow{r'}) (\overrightarrow{r}-\overrightarrow{r'})}}{|\overrightarrow{r}-\overrightarrow{r'}|}, \end{aligned}$$

where *M* is the magnetization of the sensing or the pinned layer. Likewise, the field created by the bias current ($H_{bias}^{i}$) can be calculated using^48^:

9 $$\begin{aligned} \overrightarrow{H}_{r}= \frac{1}{4 \pi } \int _V \overrightarrow{J} (\overrightarrow{r'}) \times \frac{(\overrightarrow{r}-\overrightarrow{r'})}{|\overrightarrow{r}-\overrightarrow{r'}|^3} d^3\overrightarrow{r'} , \end{aligned}$$

where *J* is the current density. The overall magnetic field is averaged at mid-thickness of the layer of interest.

## Supplementary Information

Supplementary Information.
