# Supplementary material for: Two-dimensional arrays of vertically packed spin-valves with picoTesla sensitivity at room temperature
Source: Sci Rep. 2021 Jan 8;11:215. doi: 10.1038/s41598-020-79856-0 (PMC7794481; doi:10.1038/s41598-020-79856-0)
Supplement: Supplementary file 1 — Supplementary Information. [file 41598_2020_79856_MOESM1_ESM.pdf]

Supplementary Information – Two-dimensional arrays of vertically packed spin-valves with picoTesla sensitivity at room temperature

M. Silva[1,2,*], F. Franco[1,2,+], D. C. Leitao[1,2], S. Cardoso[1,2], and P. P. Freitas[1,3]

[1]INESC - Microsistemas e Nanotecnologias, Lisboa, Portugal and
[2]Instituto Superior Tecnico (IST), Universidade de Lisboa, Lisboa, Portugal
[3]INL - International Iberian Nanotechnology Laboratory, Braga, 4715-330 Portugal
*corresponding author: msilva@inesc-mn.pt
[+]Presently at Analog Devices

Abstract

In this supplementary material details on the magnetic [M(H)] and electric behavior [R(H)] of double and N- vertically packed spin valves are described as complement of section Results and Discussion of manuscript. The influence of the spacer thickness on the M(H) curve of unpatterned thin films and on the R(H) curve of the micropatterned structures is addressed in Unpatterned thin film section. Section Micropatterned Sensor: impact of spacer thickness and physical dimensions includes considerations on strategies to optimize the response of N- vertically packed spin valves and the influence on the noise curve. Also, different array configurations are shown to improve the detectivity of the final devices.

Results and Discussion

Unpatterned thin-film

Impact of the TaOx thickness on the M(H) curve

Figure S1 shows vibrating sample magnetometry (VSM) at room temperature of a single spin valve and two packed spin valves with different TaOx spacer thickness. Figure S1(a) exhibits a full loop measured between ± 100 mT, while figs S1 (b)-(e) highlights the sensing layer region to evaluate the offset field. The lateral shift observed in the M(H) curve arises from the different surface roughness obtained depending of the TaOX thickness used , which in turn leads to different Neel coupling fields[1]. Figure S1(d) shows comparable results to those obtained for a MR (H) curve as seen in figure 2(d) of the manuscript.

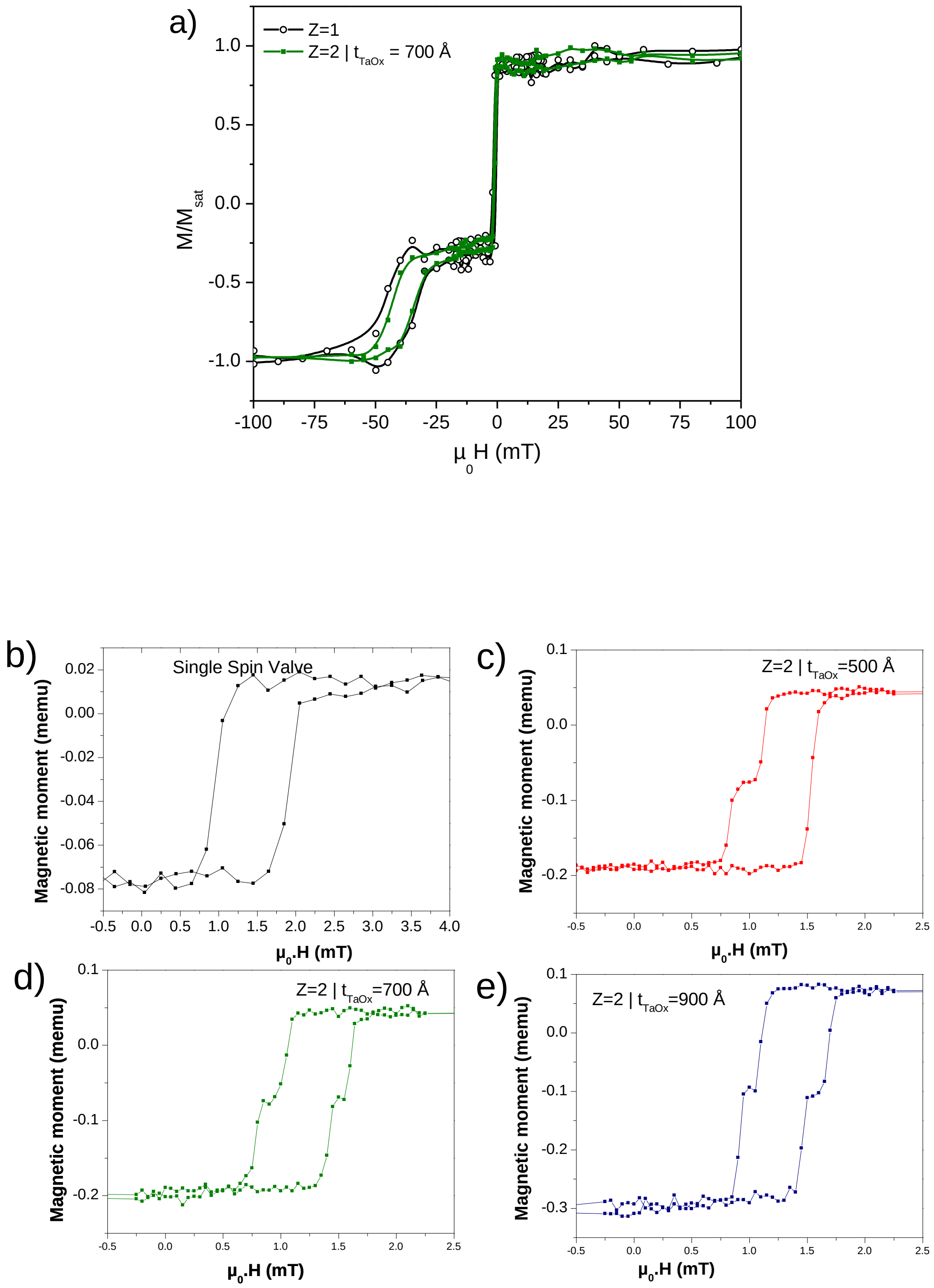

Figure S1: a) M(H) full loop of a single spin valve a double packed system with TaOx spacer thickness of 700 Å. Detail on the free layer region for (b) single spin valve and for two spin valves packed (Z=2) with TaOx spacer thickness of (c) 500 Å (d) 700 Å and (e) 900 Å.

Micropatterned Sensor: impact of spacer thickness and physical dimensions

Impact of the TaOx thickness on the R(H) curve

Figure S2 shows the R (H) curve of the micropatterned sensor for a single sensor and for a double packed spin valve (Z=2) with TaOx spacer of 700 Å and 1100 Å. The figures are the same curves shown at figure 3 (f) and (g) of the manuscript but without normalization, in order to evaluate the values of resistance. The minimum resistance of a single spin valve sensor is 698 Ω, consequently the other two systems for Z=2, should show half of the value (349 Ω) as they are connected in parallel. The experimentally obtained minimum resistance value is slightly higher than the expected, most likely due higher contact resistance, which also affects the magnetoresistance value.

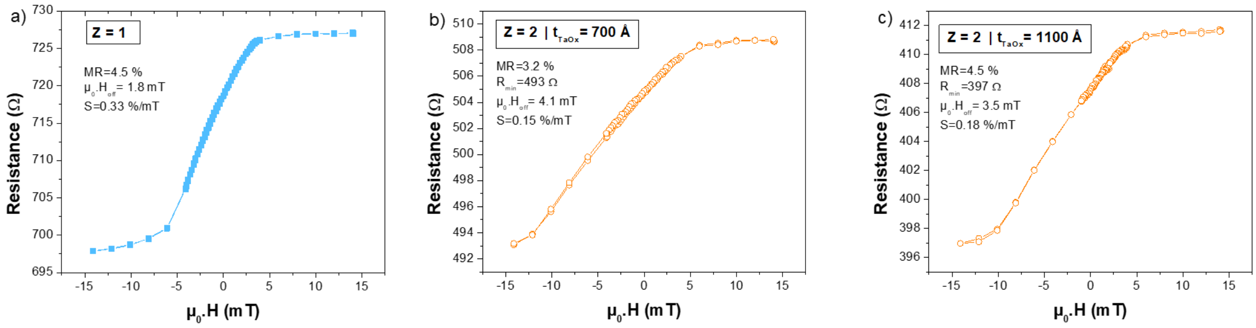

Figure S2: MR(H) curves of (a) single spin valve and *Z=2* packed spin valves with spacer thickness of (b) 700 Å (c) 1100 Å.

<u>Impact of the physical dimension on the R(H) curve</u>

The linear range of the spin valve sensors depends on the device geometry and size due to the demagnetizing field. The shape anisotropy forces the sensing layer magnetization to be along the larger dimension, while the pinned layer magnetization is fixed along the smallest dimension due to large exchange bias coupling [1]. This orthogonal configuration gives the linear transfer curve if the demagnetizing field is higher than the intrinsic anisotropy field of the sensing layer. In a packed system the spin valves are separated by an insulator hundreds of Å thick. A loss of sensitivity is visible due to the strong impact of stray fields from the neighbor sensing layer [Fig. 4 of the manuscript]. Figure S3 (a) shows the demagnetizing field (d=0) and the stray field for a spin valve width changing from *w* =2 to 100 µm and fixed length *l* = 100 µm. The stray field was calculated for different distances *d* between spin valves. The calculated stray field decreases very sharply until 10 µm with increasing width, then evolves smoothly until zero. For different distance values, the stray field shows substantial difference only until w = 5 µm. This supports the approach relying on the increase of the dimensions, instead of using a thick spacer. Figure S3 (b) shows the calculated transfer curves for Z=1 and Z=2 considering different widths and using a thin spacer layer (700 Å). By increasing *w*, the stray fields decrease and consequently the sensitivity and offset approximate a single spin valve with w = 2 um (dashed line). Based on this, the developed macrospin model was extended for a higher number of spin-valves packed vertically. The algorithm follows the same steps as shown in figure 3 (b) of the manuscript but now for higher number of Z considering all the interdependent magnetic couplings. The spacer thickness was fixed at 700 Å, and a systematic study between the number of spin-valves deposited vertically and their width was performed (see Figure 4 of the manuscript). Figure S3 (c) and (d) display the calculated curves for Z=5 and Z=10, respectively. Figures S3 (e)-(h) display a detailed comparison between experimental data and calculations showing good agreement for Z=5, while for Z= 10 larger deviations are observed due to increase in complexity in the magnetic interdependence (larger number of FM/AFM and FM/FM coupled layers), increased correlated roughness and larger probability of structural defects which may lead to domain wall pinning sites.

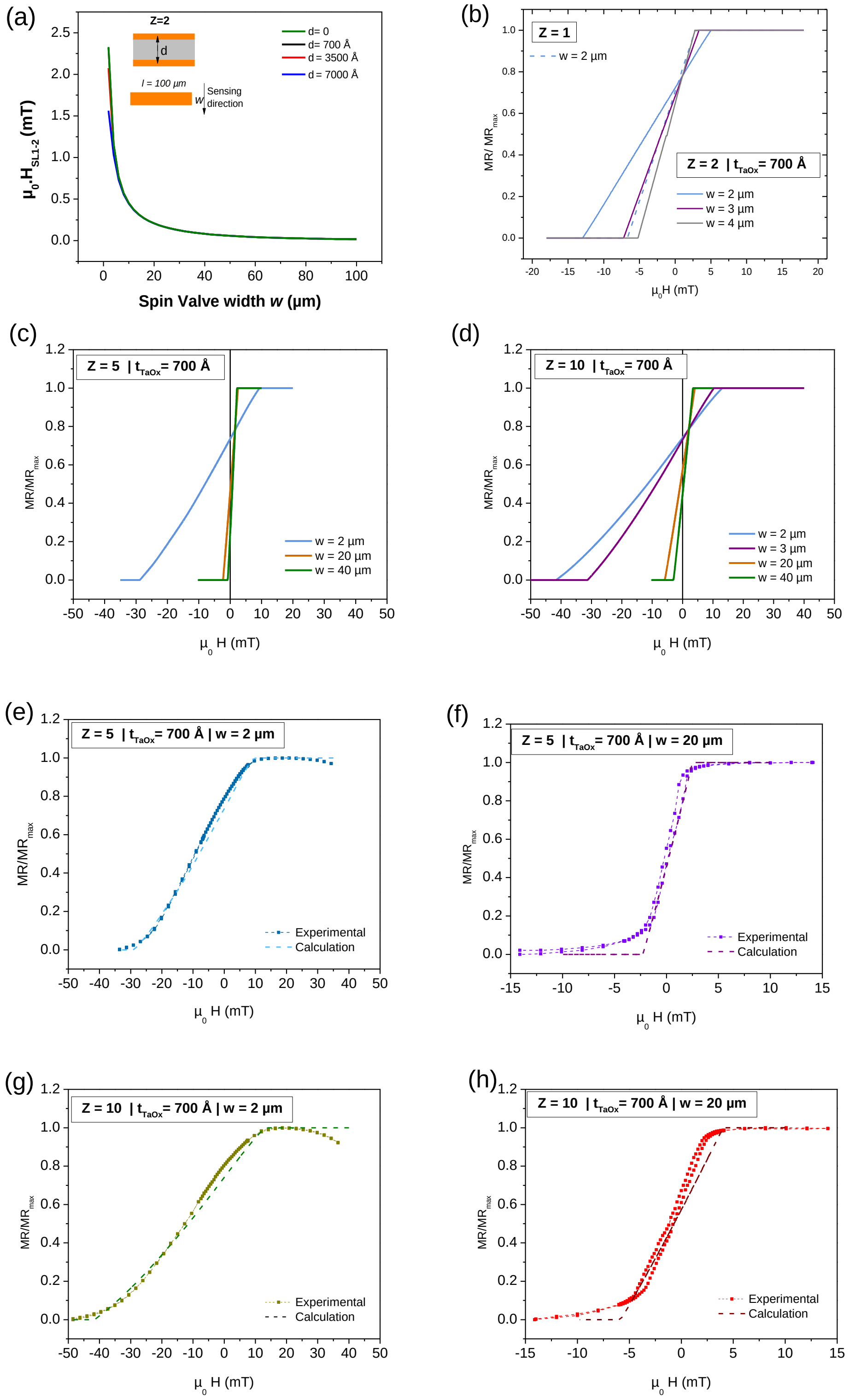

Figure S3: (a) Demagnetizing and stray fields for a Z=2 spin valve with w = 2 to 100 µm, fixed length l = 100 µm. The stray field was calculated for different distances *d* between spin valves, linked with a changing spacer thickness from 0 to 7000 Å (b) Calculated transfer curves for Z=1 and w = 2 µm (dashed line) and for Z = 2 with w = 2 to 4 µm and thin spacer thickness of 700 Å (solid line).(c) and (d) Calculated transfer curves obtained from model changing the width from 2 to 40 µm for Z=5 and Z=10, respectively. These results were obtained using the analytical model developed and the input parameters shown in table I from the manuscript (e)-(f) Comparison between experimental data and calculated curves for w = 2 µm and 20 µm for different Z.

2D arrays: reducing noise, increasing detectivity

Impact of an external transversal field on the transfer curve

As the sensor width increases also does the coercivity. To promote a hysteresis-free curve, and thus further improve the sensor output for Z= 5 and Z=10 for 20 µm < w < 100 µm, we have applied an external transversal field (H_{ext}) to the sensing direction. The magnetic field is created with a pair of external coils allowing a precise tuning of the intensity that yields optimum hysteresis-free sensors. As the external transversal field increases, a non-hysteric curve is achieved accompanied by a reduction in sensitivity and a higher offset field as shown in figure S4(a). Figure S4(b) shows the effect of the external transversal field on the offset field, sensitivity and coercivity for different sensor widths with Z=10 spin valves (similar study was done for Z=5). Regarding the coercive field is independent of the width and a reduction from 0.4 mT to 0.04 mT is visible. A sharp decrease occurs until 1.0 mT, then remaining constant in the minimum value. The sensitivity depends on the width, but the overall behavior tends to be almost constant until $\mu_0 H_{ext}$ = 1.0 mT, decreasing above this value. Concerning the offset field, a smooth increase is more visible for lower widths while for higher widths it tends to be constant. From this study, the optimum field to enhance the microstructure performance for sensing applications is achieved for an external field of 1.0 mT, where no loss on sensitivity is ensured maintaining a coercivity field lower than 0.1 mT. Such strategy can be employed as a monolithic solution, with the transverse magnetic field created on chip using integrated permanent magnet made of CoCrPt or current line loops with a compromise of losing spatial resolution [2].

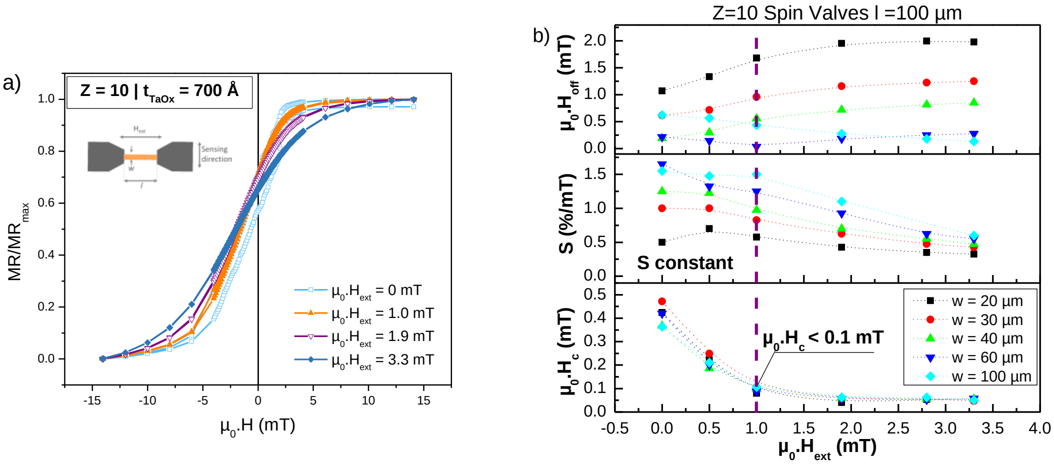

Figure S4: a) Normalized MR (H) transfer curves for external transverse field applied varying from 0 to 3.3 mT. The inset shows the direction of the external transverse field. b) Offset and coercivity field and sensitivity as function of external transverse field to sensing layer with dependence on spin valve width.

Impact of an external transverse field on the noise

Sensors with larger width present a higher coercive field due to domains formation leading higher magnetic noise [3]. By applying an external transverse field, the magnetic configurations in the sensing layer can be stabilized, improving noise.

Devices with Z=5 and w=20 µm were used to address the impact of the external transverse field on sensor noise. The noise measurement was performed at H_{app} =0 under a current bias condition of 100 mV. Figure S5 shows the noise curve of the device with (i) no field applied, (ii) $\mu_0 H_{ext}$ = 0.5 mT and (iii) $\mu_0 H_{ext}$= 1.0 mT. The maximum transverse field applied was chosen from the consideration on the previous section. No clear influence on the noise level is visible, although a small change in the field sensitivity was identified in Fig.S4.

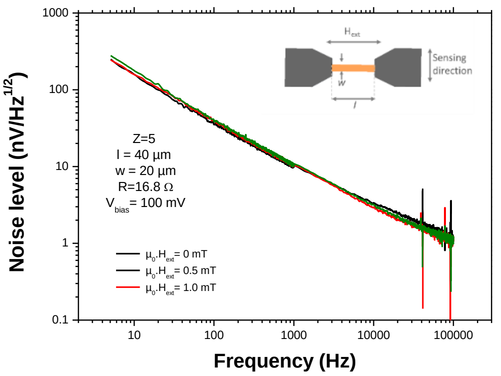

Figure S5: Noise curves for a spin valve with Z=5 spin valves and width w =20 µm at H_{app} = 0 mT, but under the effect of a transverse filed (inset).

Noise on arrays

Concerning the final device, the width of w=2 µm for single sensors and w=20 µm for sensors packed (Z=5 and 10) was chosen to compare the noise and detectivity level. The strategy used to improve the detectivity includes increasing the number of sensors by doing arrays with X elements in series, Y in parallel and Z vertically packed. The array configuration is shown in table I.

Table I: Different array configurations being Z the number of vertical spin valves, X connected in series and Y in parallel.

Total number of sensors	X	Y	Z
**100**	10	10	1
	10	2	5
	10	1	10
**200**	10	2	10
**400**	20	4	5
**500**	20	5	5
**800**	40	20	1
	20	4	10
**1000**	40	25	1
	20	10	5
**2000**	20	10	10

Figure S6 presents the noise level at 10 Hz and thermal level for different array configuration but with the same total number of sensors. All are measured with 1 V. The noise at low frequencies decreases with increasing number of sensors. This is visible because the number of sensors in parallel Y are increasing more than the number of series X leading to a reduction of the resistance. Similar behavior happens at thermal level, since the thermal noise depends only the resistance. The noise gain at low frequencies (equation 7 from manuscript) between arrays *i* and *j* is given by:

$$G = \sqrt{\frac{X_i}{X_j}\frac{Y_j}{Y_i}\frac{Z_j}{Z_i}\frac{w_j}{w_i}}.$$

Figure S6 also exhibits the footprint of arrays with the same number of elements. Array with Z=1 shows the lower spatial resolution while improved resolution is attributed to array with Z=10.

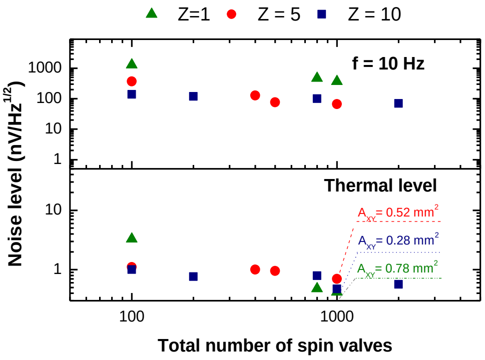

Figure S6: Noise level at 10 Hz and thermal level for arrays with different number of sensors. The arrays are composed by Z=1, 5 and 10 spin valves packed for w=2 µm (Z=1) and w = 20 µm. Also, the occupied area is specified for arrays with 1000 sensors with different Z.

<u>Detectivity on arrays</u>

The detectivity of different array configurations was calculated considering the sensitivity and noise level.

At the end, the sensitivity varies for arrays with different Z. The arrays with Z=1 show a sensitivity of 3 V/V/T and arrays with Z=5 and Z=10 display a sensitivity of 10 V/V/T and 6 V/V/T, respectively.

Devices with Z=5 show an improved detectivity, because although the noise level is higher, they have better sensitivity than Z=10. A larger width should be chosen for Z=10 to reach the same values of sensitivity and to be competitive in detectivity. The array with the same number of sensors XYZ=1000, shows a detectivity at 10 Hz of 110 nT/√Hz, 7nT/√Hz and 21 nT/√Hz for Z=1, Z=5 and Z=10, respectively.

References

[1] AV. Silva, D. C. Leitao, J. Valadeiro, J. Amaral, P. P. Freitas, and S. Cardoso, The European Physical Journal Applied Physics 72, 10601 (2015).
[2] J. P. Valadeiro, J. Amaral, D. C. Leitao, R. Ferreira, S. F. Cardoso, and P. J. Freitas, IEEE Transactions on Magnetics 51, 1 (2015)
[3] P.P. Freitas, R. Ferreira, S. Cardoso, F. Cardoso, Magnetoresistive sensors. Journal of Physics: Condensed Matter, 19(16), p.165221 (2007)
